# JAK Inhibitors: Treatment Efficacy and Safety Profile in Patients with Psoriasis

**DOI:** 10.1155/2014/283617

**Published:** 2014-05-05

**Authors:** Leeyen Hsu, April W. Armstrong

**Affiliations:** Department of Dermatology, University of Colorado at Denver, Anschutz Medical Campus, 12801 East 17th Avenue, Mail Stop 8127, Aurora, CO 80045, USA

## Abstract

Janus kinase (JAK) pathways are key mediators in the immunopathogenesis of psoriasis. Psoriasis treatment has evolved with the advent of targeted therapies, which inhibit specific components of the psoriasis proinflammatory cascade. JAK inhibitors have been studied in early phase trials for psoriasis patients, and the data are promising for these agents as potential treatment options. Tofacitinib, an oral or topically administered JAK1 and JAK3 inhibitor, and ruxolitinib, a topical JAK1 and JAK2 inhibitor, have been most extensively studied in psoriasis, and both improved clinical symptoms of psoriasis. Additional JAK1 or JAK3 inhibitors are being studied in clinical trials. In phase III trials for rheumatoid arthritis, tofacitinib was efficacious in patients with inadequate responses to tumor necrosis factor inhibitors, methotrexate monotherapy, or disease-modifying antirheumatic drugs. The results of phase III trials are pending for these therapies in psoriasis, and these agents may represent important alternatives for patients with inadequate responses to currently available agents. Further investigations with long-term clinical trials are necessary to verify their utility in psoriasis treatment and assess their safety in this patient population.

## 1. Introduction

Psoriasis is a chronic inflammatory skin disease that affects 3% of the United States population [1]. It manifests as well-demarcated, scaly patches on the skin, and it is associated with psoriatic arthritis and other comorbidities [2–4]. The choice of psoriasis treatment varies depending on the severity and extent of skin involvement. Topical therapies are reserved for mild or localized disease, whereas phototherapy and systemic therapies are used for those with moderate-to-severe disease. Limitations with extended use of traditional oral systemic therapies include suboptimal efficacy, slow onset of therapeutic effect, toxicities, and teratogenicity; these limitations have propelled the use of targeted therapies into the forefront of treatment for chronic inflammatory diseases such as psoriasis, psoriatic arthritis (PsA), and rheumatoid arthritis (RA) [5]. Over the last decade, biologic agents targeting specific components of the tumor necrosis factor (TNF-)*α* pathway have gained wide adoption for treatment of psoriasis as they achieved rapid clinical improvement with minimal side effects in multiple clinical trials and ongoing studies [6–9]. However, high costs, potential risk for adverse events, and lack of persistent effects in some patients have fueled continued search for alternative therapies that target various components of the psoriasis inflammatory cascade.

The exact mechanism of psoriasis is still not fully understood. Cytokines and growth factors such as interleukin (IL)-1, IL-6, IL-12, IL-17, IL-20, IL-23, interferon (IFN)-*γ*, and TNF-*α* within the abnormally upregulated Th1 and Th17 pathways have been implicated as key mediators in the immunopathogenesis of psoriasis by driving the activation and proliferation of epidermal keratinocytes [10–14]. After the identification of increased protein tyrosine kinase activity in immunologic diseases, therapeutic agents targeting the protein tyrosine kinases have been developed, and they are effective and well-tolerated medications [15]. The Janus family of kinases is a subset of the protein tyrosine kinases. Preclinical studies have identified a number of cytokines involved in the psoriasis inflammatory cascade that utilize the Janus family kinase (JAK) signaling pathway [16].

In this paper, we discuss the molecular pathway of the JAK-STAT signaling cascade and the mechanism of action of the JAK inhibitors. We also examine in detail the treatment efficacy and safety of the currently available JAK inhibitors for psoriasis treatment. We also briefly discuss currently available data on treatment efficacy and safety in other chronic immune-mediated diseases such as RA and ulcerative colitis (UC).

## 2. Jak-Stat Signaling Pathway

**Figure 1:**
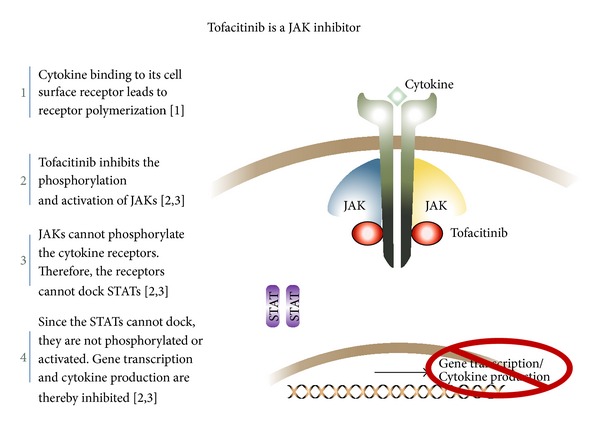
The mechanism of action of tofacitinib. JAK: Janus family kinase.

Cytokine receptor signaling involves pathways such as the JAK-STAT pathway and the MAP kinase cascade [17]. The JAK family consists of four members: JAK1, JAK2, JAK3, and TYK2. Cytokine-activated, oligomerized receptors recruit intracytoplasmic JAKs to bind in pairs. The dimerized JAKs autophosphorylate and become activated subsequently (Figure 1). The activated JAKs modify the receptors and allow STAT to bind. The activated STATs dimerize and translocate into the cell nucleus to influence DNA transcription, thus regulating gene expression [18]. The various combinations of JAK pairs recruit different STAT proteins, of which there are up to six types, and this allows for the wide range of downstream activities seen in the JAK-STAT pathways [19]. The JAK-STAT pathways activate or suppress the transcription of a wide array of genes that affect cell growth and apoptosis such as SOCS, Nmi, Bcl-XL, p21, MYC, and NOS2 [20]. However, JAKs associate with specific cytokine receptors and therefore influence different aspects of immune cell development and function. JAK1 is associated with IFN, IL-6, IL-10 receptors, and receptors containing common *γ* chains [21, 22]. JAK2 is primarily involved in hematopoietic receptors as well as IL-12 and IL-23. When dimerized with JAK1, JAK3 acts selectively on receptors containing the common *γ* chain, which include IL-2, IL-4, IL-7, IL-9, IL-15, and IL-21, which are crucial to lymphocyte function. TYK2 is associated with IFN, IL-12, and IL-23 receptors in conjunction with JAK2 [19, 23, 24]. JAK dysfunction has been associated with myeloproliferative diseases such as polycythemia vera, essential thrombocytopenia, and myelofibrosis as well as inherited immunodeficiencies including severe combined immunodeficiency and hyperimmunoglobulin E syndrome [25]. Dysregulation of JAK signaling has also been identified in multiple autoimmune disorders [21]. Due to their ability to selectively modulate immune function, targeted JAK inhibitors are attractive candidates for immune-mediated diseases.

## 3. Mechanism of Action of JAK Inhibitors

Tofacitinib, a JAK1 and JAK3 inhibitor, and ruxolitinib, a JAK1 and JAK2 inhibitor, are the most extensively studied JAK inhibitors in psoriasis [23]. Blocking these upstream components of the proinflammatory signaling pathways results in alterations in the immune response and suppresses the abnormal activation of the inflammatory cascade in diseases such as psoriasis (Figure 1) [26]. In murine models, tofacitinib suppressed the expression of IL-23 receptors, IL-17A, IL-17F, and IL-22 when T cells were stimulated with proinflammatory cytokines such as IL-6 and IL-23 [27]. Inhibition of IL-23 receptor expression results in suppression of Th17 cell differentiation, which is a key driving factor in the pathogenesis of psoriasis [21]. Additionally, tofacitinib's inhibition of IL-15 may play an important role in treating psoriasis as IL-15 is highly expressed with enhanced binding activity in psoriatic lesions and associated with increased resistance to keratinocyte apoptosis [28]. Ruxolitinib acts by inhibiting JAK1 and JAK2 pathways through blocking STAT3 phosphorylation due to IL-6, IL-12, or IL-23, resulting in the suppression of pathogenic Th17 cells differentiation [29–31]. This leads to a dose-dependent decrease in production of IL-17, IL-20, and IL-22. Additionally, the suppression of STAT3 phosphorylation reduces IFN-*γ* expression, which is one of the most potent activators of keratinocyte proinflammatory function. In a study by Fridman et al., topical application of ruxolitinib in murine models reduced lymphocytic infiltration, inhibited acanthosis, and suppressed production of IL-22 induced by intradermal IL-23 [30]. JAK inhibitors act on multiple cell lines that contribute to the clinical manifestations of psoriasis [14, 32].

## 4. Tofacitinib in Psoriasis

Tofacitinib, a JAK1 and JAK3 inhibitor, has undergone the most extensive clinical studies of JAK inhibitors in psoriasis treatment [33–37]. In a phase I dose-escalation trial by Boy et al., a 14-day course of oral tofacitinib 5 mg twice daily b.i.d., 10 mg b.i.d., 20 mg b.i.d., 30 mg b.i.d., 50 mg b.i.d., and 60 mg once daily (q.d.) was administered to 59 patients with mild-to-moderate psoriasis [33]. On day 14, the investigators found that every tofacitinib dosage group except 5 mg b.i.d. had dose-dependent improvement in the least squares mean (LSM) of percentage change in the psoriatic lesion severity sum (PLSS) score compared to the placebo group (*P* < 0.01) [33]. Three of the eight patients receiving tofacitinib 50 mg had PLSS scores of 0 by day 14 from baseline scores of 4–6. On day 14, the physician's global assessment (PGA) score improvement, defined as “almost clear” or “clear” and a ≥2-point PGA score improvement, in patients receiving 50 mg b.i.d. were higher than in the placebo group (*P* < 0.05). Of the skin biopsy samples obtained, marked histological improvements were noted in patients receiving a dosage of 30 mg b.i.d. when compared to their baseline, while lesional biopsies from the placebo group showed minimal or no change compared to baseline. Of the 16 adverse events in 10 patients within this study, headaches (*n* = 5) and nausea (*n* = 3) were most common, and all suspected treatment-related adverse effects were considered mild. One patient had moderate progression of psoriasis. Of the laboratory studies conducted, Boy et al. reported elevated total cholesterol, low-density lipoprotein cholesterol, and triglyceride in the treatment groups when compared to the placebo group [33].

In a 12-week phase IIb study, Papp et al. described the efficacy and safety of oral tofacitinib 2 mg b.i.d., 5 mg b.i.d., or 15 mg b.i.d. in 197 moderate-to-severe psoriasis patients [34]. Papp et al. reported psoriasis area and severity index (PASI) 75 response rates of 25.0% (2 mg; *P* < 0.001), 40.8% (5 mg; *P* < 0.0001), and 66.7% (15 mg; *P* < 0.0001) versus 2.0% in the placebo group at week 12 [34]. More PASI 75 responders were observed in all treatment groups as early as week 4 and maintained through week 12 compared to placebo patients (*P* < 0.05 to *P* < 0.001). Upper respiratory tract infections, nasopharyngitis, and headache were the most common adverse effects reported by the patient cohort. Three patients experienced five serious adverse events including angina pectoris, pyelonephritis, urosepsis, and atrial fibrillation. However, the study did not specify whether these events were treatment related. Discontinuation from the study was reported in 2.0%, 4.1%, and 6.1% of patients in the 2, 5, and 15 mg b.i.d. groups versus 6.0% of patients in the placebo group. Serum creatinine increased (mean 0.04 mg dL^−1^) in the 15 mg b.i.d. group at week 12 when compared to their baseline. One case of alanine aminotransferase elevated greater than 2.5 times the upper limit of normal was documented in the 15 mg b.i.d. group. Tofacitinib treatment was associated with mild, dose-dependent decreases in hemoglobin of 0.15, 0.20, 0.14, and 0.71 g dL^−1^ for placebo and tofacitinib 2, 5, and 15 mg b.i.d. groups, respectively, at week 12 [34, 38]. Additionally, mean absolute neutrophil counts decreased at higher doses of tofacitinib with a maximum mean decrease of 0.9 × 10^3^ mm^−3^ in patients receiving 15 mg b.i.d. at week 4. However, these values began to return to baseline values from weeks 4 to 8 [34, 38].

In this same study cohort, Mamolo et al. described the patient-reported outcomes of these 197 patients with moderate-to-severe psoriasis through six patient questionnaires [36]. At week 12, the authors reported greater LSM changes from baseline for the dermatology life quality index, itch severity score, and short form-36 questionnaire mental component for all active drug arms versus placebo (*P* < 0.05) [36]. A total of 35.1%, 38.5%, and 74.4% of patients in the 2, 5, and 15 mg groups, respectively, reported “clear” or “almost clear” on the patient global assessment of psoriasis versus 2.9% for the placebo group (*P* < 0.0001 for all doses) [36]. Tofacitinib improved both physician- and patient-reported outcomes.

Tofacitinib has also been utilized as a topical formulation. In a vehicle-controlled phase IIa trial studying a topical tofacitinib ointment formulation, Ports et al. reported the data on 71 mild-to-moderate psoriasis patients treated with tofacitinib 2% ointment 1 b.i.d. versus tofacitinib 2% ointment 2 b.i.d. for 2 weeks to a single, fixed 300 cm^2^ treatment area containing a target plaque [35]. This study noted improvement in the target plaque severity score (TPSS) at week 4 for ointment 1 (LSM-54.4%) versus vehicle 1 (LSM-41.5%; one sided 90% upper confidence limit <0) but not for ointment 2 (LSM-24.2%) versus vehicle 2 (LSM-17.2%; one sided 90% upper confidence limit >0). Systemic concentrations (>0.100 ng mL^−1^) were detected in 12 (60%) of 20 patients receiving ointment 1 for at least one time point compared to 6 (26%) of 23 patients receiving ointment 2. However, these serologic levels were 40-fold lower than the systemic concentration achieved at the lowest oral dose tested (2 mg b.i.d.) [34]. A total of 25 out of 71 patients reported adverse effects; all of them were categorized as mild or moderate. Nasopharyngitis (*n* = 4) and urinary tract infections (*n* = 3) were the most common [35]. Clinical trials testing tofacitinib administered either orally or topically for psoriasis have shown statistically significant symptom improvement in patients with psoriasis when compared to their placebo counterparts.

There are multiple phase III trials (NCT01186744, NCT01276639, NCT01309737, NCT01163253, and NCT01815424) studying the efficacy and safety of tofacitinib in psoriasis patients [39]. One phase III trial (NCT01241591) has compared oral tofacitinib 5 mg or 10 mg b.i.d. versus etanercept 50 mg twice weekly for 12 weeks for patients with moderate-to-severe psoriasis, and the results are pending at the time of writing of this paper. Two phase IIa trials (NCT01246583 and NCT00678561) and one phase IIb trial (NCT01831466) of tofacitinib ointments are under way as well. Additional phase III studies (NCT01519089, NCT01976364, and NCT01877668) are examining the efficacy and safety of tofacitinib in patients with PsA. One phase III study (NCT01882439) has begun recruiting participants for tofacitinib in PsA patients with inadequate response to at least one TNF inhibitor.

## 5. Ruxolitinib in Psoriasis

Ruxolitinib, a JAK1 and JAK2 inhibitor, has primarily been studied as a topical ointment for mild-to-moderate psoriasis, and it has been compared to other topical therapies, which include topical steroids and topical calcipotriene [19, 40]. In a phase II study, Punwani et al. described the treatment of 28 patients with limited psoriasis (<20% body surface area) who were divided into 5 treatment groups: ruxolitinib 0.5% cream q.d. versus vehicle (Group 1), ruxolitinib 1.0% cream q.d. versus vehicle (Group 2), ruxolitinib 1.5% cream b.i.d. versus vehicle (Group 3), ruxolitinib 1.5% cream versus calcipotriene 0.005% cream b.i.d. (Group 4), or ruxolitinib 1.5% cream versus betamethasone dipropionate 0.05% cream b.i.d. for 28 days (Group 5) [40]. The lesions were evaluated by the total lesion score (0–12), which was a composite of the target lesion scores for erythema, scaling, and thickness, each rated on a scale of 0 to 4. On day 28, the total lesion scores were relatively similar to the vehicles in patients receiving ruxolitinib 0.5% cream, whereas the total lesion scores decreased by 53% and 54% in patients receiving ruxolitinib 1.0% cream q.d. and ruxolitinib 1.5% cream b.i.d., respectively, versus 32% and 32% in patients in their respective vehicle cohorts (*P* = 0.033 and 0.056, resp.). The authors also noted that the onset of effect and efficacy of ruxolitinib 1.5% cream b.i.d. was comparable to that of topical calcipotriene and betamethasone dipropionate. Mean plasma concentrations of ruxolitinib for the 0.5%, 1.0%, and 1.5% cream were 0.32 ± 0.40, 0.96 ± 0.82, and 2.10 ± 1.78 nmol L^−1^, all of which were well below the plasma concentration determined to be pharmacologically active, which suggests that the topical ruxolitinib preparations are unlikely to cause systemic adverse effects. Adverse effects including stinging, itching, irritation, pain, dryness, exfoliation, and/or redness at the application site were all mild and reported in 6 (20%) of ruxolitinib-treated lesions, 5 (28%) of the vehicle-treated lesions, 2 (33%) of the calcipotriene-treated lesions, and 2 (40%) of the betamethasone-treated lesions [40].

In a study referenced by Ortiz-Ibanez et al., 200 mild-to-moderate psoriasis patients were divided into 3 treatment groups receiving topical ruxolitinib at doses of 0.5%, 1%, and 1.5% cream for 3 months in a phase IIb vehicle-controlled trial (primary publication of the study results are not yet in the literature) [19]. In the 1% cream cohort, mean PASI improvement was 40% versus 1% with placebo. Local irritation was cited as the most frequent adverse effect, and respiratory infections were reported in 6.7% of the patients receiving ruxolitinib 1.0% cream versus 2% of patients in the placebo group [19]. Of the early clinical studies available, ruxolitinib may be a promising agent for topical treatment of psoriasis. A clinical study of ruxolitinib in a phase trial (NCT00617994) is underway.

## 6. Other Jak Inhibitors

There are two additional JAK inhibitors undergoing investigation for the treatment of moderate-to-severe psoriasis [19]. ASP015 K, a selective JAK3 inhibitor, has undergone a phase IIa dose escalation study (NCT01096862) in a cohort of 124 patients with moderate-to-severe psoriasis (see http://www.clinicaltrials.gov). INCB-28050/LY3009104, a JAK1 and JAK3 inhibitor, is being examined in a phase IIb dose ranging study (NCT01490632) in 240 moderate-to-severe psoriasis patients (see http://www.clinicaltrials.gov). Additional JAK inhibitors have been developed as potential therapies for psoriasis such as VX-509 and R-348, but there are no documented clinical trials examining these agents in psoriasis patients [19].

## 7. Discussion

JAK inhibitors in early phase trials produced significant clinical improvement in psoriasis when compared to placebo groups. These findings show that cytokine signaling through the JAK pathway is an important driver in the pathogenesis of psoriasis [16]. The JAK pathway is involved in the intracellular signaling that affects various cytokines, which propagate a wide range of downstream effects. The JAK inhibitors currently under investigation target one or more members of the JAK family. Their mechanism of action involves targeted inhibition of both upstream and downstream components of proinflammatory pathways in psoriasis, and these medications represent a promising class of agents for the treatment of psoriasis.

Tofacitinib and ruxolitinib are the two JAK inhibitors that have been most studied in psoriasis. Tofacitinib, studied as both an oral and topical administration, has undergone the most extensive clinical testing thus far with ongoing phase III clinical trials likely completed at the time of publication of this manuscript. Phase I and II clinical trials on tofacitinib, a JAK1 and JAK3 inhibitor, reported dose-dependent improvement in patients with psoriasis when compared to the placebo groups [33–36]. Ruxolitinib, a JAK1 and JAK2 inhibitor, has exclusively been studied as a topical formulation for the treatment of mild-to-moderate psoriasis. Studies have reported that ruxolitinib is an efficacious topical therapy with limited systemic exposure. The plasma concentrations of ruxolitinib in patients receiving topical medication were less than 1% of the concentrations required for systemic activity in healthy volunteers, suggesting that ruxolitinib locally inhibits the propagating factors of psoriasis rather than through systemic effects [40]. Considering the issues associated with targeted agents requiring invasive administration in psoriasis, the noninvasive administration route of JAK inhibitors is a favorable attribute of these drugs. The data discussed herein suggest that JAK inhibitors represent an important choice in the current armamentarium of psoriasis therapies.

Due to the wide array of downstream targets that JAK inhibition affects, concerns have been raised that JAK inhibitors may impair the body's ability to fight infections as well as modify hematopoietic development and function [25]. Although the safety profiles of tofacitinib and ruxolitinib were acceptable in the early phase trials, there is still concern for unknown long-term side effects with these medications [33–35, 38, 40]. Of the studies investigating tofacitinib, upper respiratory tract infections, headaches, and mild nausea were cited as the most common adverse effects experienced by patients [33, 34]. Papp et al. noted that the rate and type of adverse effects between the treatment and placebo groups were relatively similar [34]. The safety of tofacitinib has been more extensively studied in phase trials for RA patients, and the side effect profiles were similar to those reported in psoriasis patients [39]. Based on murine models, there has also been concern for possible reactivation of tuberculosis and other latent infections with the use of tofacitinib [39, 41]. Although no cases of tuberculosis were reported in psoriasis patients treated with tofacitinib, cases of tuberculosis have been reported in phase trials for RA patients [39]. Tofacitinib treatment was associated with dose-dependent decreases in mean neutrophil counts and hemoglobin. However, these changes did not require intervention and the blood counts normalized during the treatment period [38]. Increases in mean low-density lipoprotein cholesterol, high-density lipoprotein cholesterol, total cholesterol, triglycerides, and transaminase levels were also observed in selected patients treated with tofacitinib [33, 34]. The manifestation of these serum changes is unclear, and further investigation is needed to determine whether any intervention is required. Patients treated with ruxolitinib primarily experienced localized adverse effects [40]. This was likely due to the minimal systemic absorption based on the mean serum drug levels. Punwani et al. also noted that patients treated with ruxolitinib ointment had fewer adverse effects than patients treated with the vehicle, calcipotriene, or betamethasone applications, supporting the safety profile of ruxolitinib [40]. Although the safety profiles of both tofacitinib and ruxolitinib appear promising with short-term use, the results must be interpreted with caution as these findings cannot confirm their safety with long-term use. Therefore, further studies are needed to determine their long-term safety profile. Findings from trials examining JAK inhibitors in other immune-mediated diseases may guide our understanding of these agents in psoriasis patients.

JAK inhibitors have been studied extensively in other chronic inflammatory conditions such as RA and UC [42–50]. Tofacitinib has been the most extensively studied JAK inhibitor in the realm of inflammatory diseases, specifically in RA. It is an efficacious treatment option either as monotherapy or in combination with methotrexate in patients with moderate-to-severe RA [42–44, 46, 47, 49, 50]. Of particular interest, tofacitinib achieved significant clinical response in patients who were refractory to treatments such as methotrexate monotherapy, disease-modifying antirheumatic drugs (DMARDs), or TNF inhibitors in phase II and phase III clinical studies [42, 43, 46, 47, 50]. In a 12-month phase III trial, van Vollenhoven et al. reported the treatment of 717 RA patients on stable doses of methotrexate and receiving tofacitinib 5 mg b.i.d., tofacitinib 10 mg b.i.d., adalimumab 40 mg once every two weeks, or placebo [50]. The authors found that the clinical response rates were better than placebo (28.3%) in patients receiving tofacitinib 5 mg (51.5%), 10 mg (52.6%), and adalimumab (47.2%; *P* < 0.001 for all comparisons), indicating not only does tofacitinib produce clinically significant improvement in RA symptoms but also it achieves a numerically similar response rate as adalimumab [41, 50]. Tofacitinib is being studied in clinical trials for the treatment of psoriatic arthritis, ankylosing spondylitis, atopic dermatitis, and keratoconjunctivitis sicca. Clinical trials on ruxolitinib in patients with RA and severe alopecia areata are underway. Clinical experience with these therapeutic options in inflammatory diseases such as RA and UC has guided the way for their potential use as agents for psoriasis.

## 8. Conclusions

JAK inhibitors are new, promising therapies in psoriasis, and they have different safety profiles from the existing traditional oral systemic medications or biologic medications. Of the JAK inhibitors studied for psoriasis, tofacitinib has been most extensively studied, and phase III study results (NCT01241591) comparing tofacitinib to etanercept are pending [51]. Ruxolitinib, ASP015 K, and LY3009104 are among the other JAK inhibitors being studied for clinical use. Overall, JAK inhibitors represent a new class of efficacious treatments to reduce disease severity and improve quality of life among psoriasis patients.

## Abbreviations

PsA: Psoriatic arthritis
RA: Rheumatoid arthritis
UC: Ulcerative colitis
IL: Interleukin
IFN: Interferon
TNF: Tumor necrosis factor
JAK: Janus kinase
b.i.d.: Twice daily
q.d.: Once daily
PGA: Physician's global assessment
LSM: Least squares mean
PLSS: Psoriatic lesion severity sum
PASI: Psoriasis area and severity index
TPSS: Target plaque severity score
DMARDs: Disease-modifying antirheumatic drugs.
